# Case of spontaneous regression of carotid body tumor in a SDHD mutant: a discussion on potential mechanisms based on a review of the literature

**DOI:** 10.1186/1477-7819-10-218

**Published:** 2012-10-19

**Authors:** Sebastiaan Hammer, Jeroen C Jansen, Eleonora PM van der Kleij-Corssmit, Frederik J Hes, Mark C Kruit

**Affiliations:** 1 Departments of Radiology, Leiden University Medical Center, Albinusdreef 2, Leiden, ZA, 2333, The Netherlands; 2 Departments of Head and Neck Surgery, Leiden University Medical Center, Albinusdreef 2, Leiden, ZA, 2333, The Netherlands; 3 Departments of Endocrinology, Leiden University Medical Center, Albinusdreef 2, Leiden, ZA, 2333, The Netherlands; 4 Departments of Human and Clinical Genetics, Leiden University Medical Center, Albinusdreef 2, Leiden, ZA, 2333, The Netherlands

**Keywords:** Tumor regression, Head and neck paragangliomas, Spontaneous involution, Surgical resection

## Abstract

**Background:**

Head and neck paragangliomas are tumors associated with the parasympathetic nerve system and typically show an indolent growth pattern. Therefore a conservative management strategy is considered in selected cases.

**Methods and results:**

We present a case of a female patient who presented in 2003 with bilateral carotid body tumors and a tympanic tumor, associated with a mutation in the *succinate dehydrogenase* -sub-unit-D (SDHD). She was operated on the right carotid body tumor and the tympanic tumor. Thereafter the follow-up was performed with MR examinations at 2-year intervals. After an initial stable phase, over the last 3 years a spontaneous near-total regression of the contralateral carotid body tumor was observed, with only subtle rest-abnormalities visible in 2011.

**Conclusions:**

The present case underlines the indolent growth pattern of head and neck paragangliomas and for the first time describes a rare manifestation of spontaneous regression of a carotid body tumor. The literature was reviewed to discuss this phenomenon.

## Background

Head and neck paragangliomas (HNPGL) are usually benign, slow-growing tumors associated with the parasympathetic nerve system. Common sites include the carotid body, the temporal bone, and the vagal body [1]. The majority of patients presents as apparently sporadic patients, whereas 10% to 20% of patients report a positive family history [2,3]. Multiple paragangliomas may occur in up to 40% of patients [4]. In the Netherlands the majority of cases are related to mutations in the gene encoding for the oxidative chain protein *succinate dehydrogenase*-sub-unit-D (*SDHD)*[5]. In the neck, a paraganglioma presents as a non-tender mass or as a cause of lower cranial nerve palsy due to local compression. In the temporal bone, the first symptom of paragangliomas is usually pulsating tinnitus, due to the hypervascular nature of the tumor. In follow-up about 60% of the HNPGL do not exhibit growth, and if they do it typically is in an indolent growth pattern with a median tumor double time of 4.2 years [6]. Diagnosis is generally made through a combination of clinical findings and magnetic resonance imaging (MRI) studies. Treatment considerations include the nature of the tumor (malignant or benign), the location, vasculature encasement, the extent, and growth rate [6,7]. Treatment modalities include surgery or radiotherapy/surgery; treatment of choice for carotid body tumors is surgical resection. However, because of the slow growth rate and potential treatment-related injury to the neighboring vessels and nerves, a conservative management strategy (wait-and-scan policy) should be considered [6].

## Case presentation

In 2003 a 32-year-old female patient was referred to our institution after magnetic resonance imaging (MRI) examination in a regional hospital had revealed bilateral carotid body tumors. She had discovered a swelling in the right neck since 6 months before, and only after questioning mentioned a long-existing pulsatile tinnitus on the right side. The family history for paragangliomas was positive: her father and uncle were affected.

Physical examination revealed a small reddish tumor in the right middle ear, with positive Brown’s sign and a tumor in the right neck at the level of the hyoid bone; on the left side there was no palpable lesion. The lower cranial nerves were intact and the hearing was normal. In general examination, the patient was all-over obese (weight 121 kg, length 1.75 m, body mass index 39.5), blood pressure was 130/70 mm Hg, pulse rate 80/min. There were no other abnormalities. Urinary excretion (24-h sample) of catecholamines was repeatedly normal. Germline mutation analysis in DNA from a blood sample demonstrated a mutation in SDH*D* (P-Asp92Tyr).

**Figure 1:**
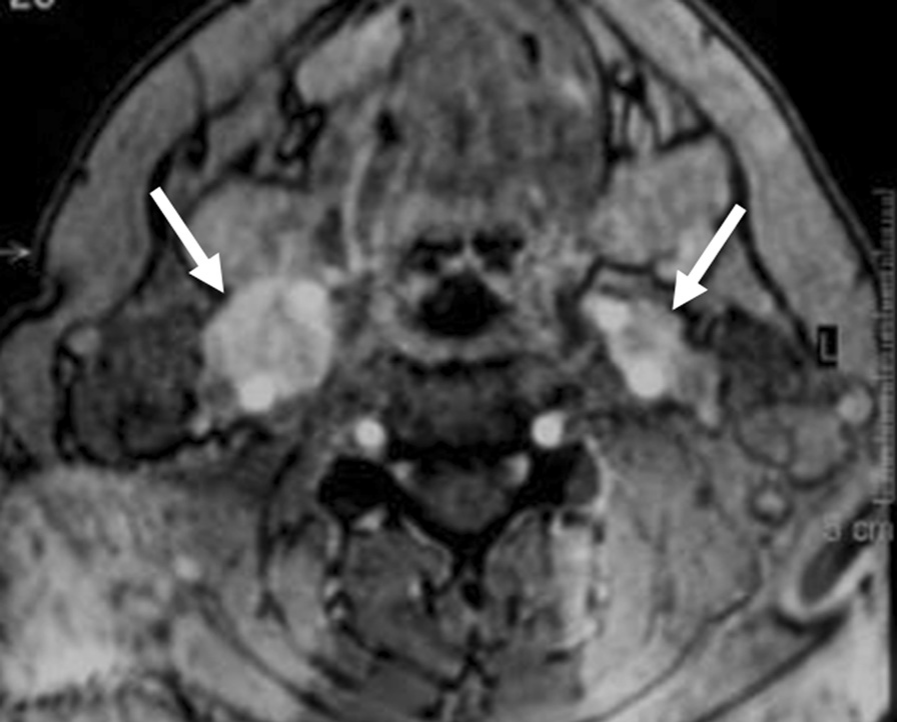
Transverse gadolinium-enhanced 3D time of flight magnetic resonance angiography images at baseline in 2004 show the bilateral carotid body tumors (arrows).

MRI including a gadolinium-enhanced 3D time-of-flight MR angiography sequence [8] was acquired at our institution in April 2004, and revealed hypervascular enhancing lesions in the carotid bifurcation bilaterally, consistent with carotid body tumors. The maximum transverse diameter was 23 mm on the right and 12 mm on the left (Figure 1). A tympanic tumor was identified on the right side.

The right carotid body tumor was surgically resected in 2004, followed by extirpation of the tympanic tumor in 2005. No radiotherapy was applied. There were no complications from the surgical procedures. In the postoperative phase a severe obstructive sleep apnea syndrome (OSAS) was diagnosed, for which she started continuous positive airway pressure (CPAP) therapy. In 2005 she started thyroid hormone suppletion for Hashimoto disease. Between 2005 and 2008, the patient intentionally lost weight from 136 kg to 93 kg, because of a pregnancy wish which was eventually unsuccessful. During follow-up the patient regained bodyweight in 2010 to 129 kg. In 2011 she suffered anamnestically from a transient ischemic attack (with no abnormalities on imaging studies), after which she started statin treatment and therapy with carbaselate calcium and dipyridamole.

**Figure 2:**
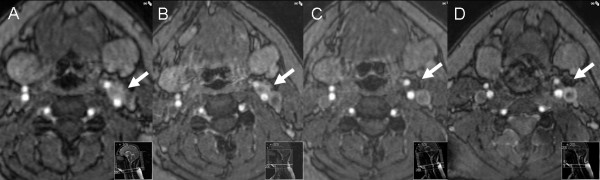
**Transverse gadolinium-enhanced 3D time-of-flight magnetic resonance angiography images at subsequent intervals showing the carotid body tumor on the left in 2006 (A), unchanged in 2008 (B), inhomogeneous ring-like enhancement in 2010 (C), and further regression in 2011 (D).** On the image only a subtle linear enhancement is noted, no evidence of an enhancing mass could be detected.

Follow-up MRI was performed with 2-year intervals (Figure 2), and showed no evidence of recurrence on the operated sites. Between 2004 and 2008 there were no changes in size and enhancement pattern of the left carotid body tumor and therefore surgical resection was considered unnecessary. Unexpectedly, in 2010, the tumor was significantly smaller, and showed reduced enhancement in the center of the lesion, consistent with necrosis. Follow-up examinations in June 2011 and September 2011 showed nearly complete regression, leaving only minimal rest-abnormalities at the site of the previous tumor. Other affected family members showed no signs of regression during follow-up.

## Discussion

We describe a patient with hereditary bilateral head and neck paragangliomas who underwent surgical tumor removal of a right carotid body tumor and a right tympanic tumor. During the 7-year follow-up, spontaneous regression of the left carotid body tumor was noted, after an initial period without any change in tumor size. To the best of our knowledge, spontaneous involution of head and neck paragangliomas has not been reported before. In our referral center for paragangliomas, the present case with spontaneous involution represents the first from an estimated total of more than 400 followed cases.

Spontaneous regression of tumors in general is a rare phenomenon, although numerous cases and case series can be found in the literature. Spontaneous remission of tumors with a common neural crest embryological origin as paragangliomas has been described as well. Regression of pheochromocytomas after initial presentation with hypertensive crises or shock has been reported [9-11], as well as spontaneous regression [12], Another known entity is spontaneous regression in a subset of pediatric neuroblastomas, especially those detected with mass screening and with biologically favorable characteristics [13,14].

### Mechanism

The mechanism that accounts for the regression in the present case can only be speculated upon. Below we discuss the general tumor regression hypotheses, including biological (genetic, immunological), hormonal (such as contraceptive use), vascular (vascular insufficiency/tumor necrosis/spontaneous intratumoral vascular thrombosis), and operative mechanisms [15].

Biological mechanisms related to tumor regression include genetic instability (telomerase inhibition) and programmed cell death, which has been described for neuroblastomas [16,17]. The specific *SDHD* mutation in the present case (p.Asp92Tyr), however, is relatively common - as it is found in almost 70% of 690 Dutch SDH-gene mutation carriers [5] and has not been reported to be associated with spontaneous tumor regression before.

Although changes in body weight may go with changes in catecholamine levels, we found no evidence for such changes in our patient, neither after the start of CPAP treatment for OSAS. Therefore a hormonal mechanism for tumor regression seems unlikely.

Vascular mechanisms may be due to changes in tumor angiostructure, and have been reported in spontaneous regressed (biologically favorable) neuroblastomas [18]. Such vascular mechanism could have explained the regression in the present case, although we have no supportive biomarkers for this hypothesis.

Operative mechanisms include regression of a tumor after biopsy, which has been described in patients with Merkel cell tumors [19,20], and involution of metastases after resection of a primary tumor, recently described in a patient with lung metastases of hepatocellular carcinoma [21]. Another example comes from a patient with three hemangioblastomas (not related to Von Hippel-Lindau disease), of which two regressed completely 6 months after surgical resection of the first tumor [22]. The authors hypothesized that the resected tumor may have been supportive for the existence of the other two. Furthermore, in two patients with neurofibromatosis type 2 and bilateral vestibular schwannomas, spontaneous regression after resection of the contralateral tumor was reported [23]. In this paper, one patient showed an initial increase in tumor size, directly after resection of the contralateral schwannoma. Afterwards, a gradual decrease in tumor size was objectified from 6 months postoperatively. Tumor size increased initially in the second patient as well, whereas a decrease in tumor size was noted from approximately 60 months [23]. A similar mechanism may explain the regression of the left carotid body tumor after initial resection of the carotid body tumor on the right in our case.

Although the present case describes spontaneous involution, surgical resection is the favored treatment in carotid body tumors. Nevertheless, resection is associated with complications such as cranial nerve impairment, stroke, or partial scarification of the carotid arteries [24].

## Conclusions

In conclusion, the present case underlines the indolent growth pattern of head and neck paragangliomas and for the first time describes a rare manifestation of involution of a carotid body tumor.

## Consent

Written informed consent was obtained from the patient for publication of this case report and any accompanying images. A copy of the written consent is available for review by the Editor-in-Chief of this journal.

## Abbreviations

CPAP: Continuous positive airway pressure; HNPGL: Head and neck paragangliomas; MRI: Magnetic resonance imaging; OSAS: Obstructive sleep apnea syndrome; SDHD: *Succinate dehydrogenase*-sub-unit-D.

## Competing interest

All authors declare no competing interest.

## Authors’ contributions

SH and MK carried out the MR examinations and drafted the manuscript. FH performed genetic analysis and drafted the manuscript. JJ and EC performed physical examinations and patient follow-up and drafted the manuscript. All authors read and approved the final manuscript.
